# Eicosanoids in Cancer: New Roles in Immunoregulation

**DOI:** 10.3389/fphar.2020.595498

**Published:** 2020-11-18

**Authors:** Amber M. Johnson, Emily K. Kleczko, Raphael A. Nemenoff

**Affiliations:** Department of Medicine, University of Colorado Anschutz Medical Campus, Aurora, CO, United States

**Keywords:** cancer, eicosanoids, Tumor microenvironment, immunosuppression, T cells

## Abstract

Eicosanoids represent a family of active biolipids derived from arachidonic acid primarily through the action of cytosolic phospholipase A2-α. Three major downstream pathways have been defined: the cyclooxygenase (COX) pathway which produces prostaglandins and thromboxanes; the 5-lipoxygenase pathway (5-LO), which produces leukotrienes, lipoxins and hydroxyeicosatetraenoic acids, and the cytochrome P450 pathway which produces epoxygenated fatty acids. In general, these lipid mediators are released and act in an autocrine or paracrine fashion through binding to cell surface receptors. The pattern of eicosanoid production is cell specific, and is determined by cell-specific expression of downstream synthases. Increased eicosanoid production is associated with inflammation and a panel of specific inhibitors have been developed designated non-steroidal anti-inflammatory drugs. In cancer, eicosanoids are produced both by tumor cells as well as cells of the tumor microenvironment. Earlier studies demonstrated that prostaglandin E2, produced through the action of COX-2, promoted cancer cell proliferation and metastasis in multiple cancers. This resulted in the development of COX-2 inhibitors as potential therapeutic agents. However, cardiac toxicities associated with these agents limited their use as therapeutic agents. The advent of immunotherapy, especially the use of immune checkpoint inhibitors has revolutionized cancer treatment in multiple malignancies. However, the majority of patients do not respond to these agents as monotherapy, leading to intense investigation of other pathways mediating immunosuppression in order to develop rational combination therapies. Recent data have indicated that PGE2 has immunosuppressive activity, leading to renewed interest in targeting this pathway. However, little is known regarding the role of other eicosanoids in modulating the tumor microenvironment, and regulating anti-tumor immunity. This article reviews the role of eicosanoids in cancer, with a focus on their role in modulating the tumor microenvironment. While the role of PGE2 will be discussed, data implicating other eicosanoids, especially products produced through the lipoxygenase and cytochrome P450 pathway will be examined. The existence of small molecular inhibitors and activators of eicosanoid pathways such as specific receptor blockers make them attractive candidates for therapeutic trials, especially in combination with novel immunotherapies such as immune checkpoint inhibitors.

## Introduction

Eicosanoids represent a family of lipid signaling molecules which are produced through the release of arachidonic acids from membrane phospholipids, and subsequent production through a series of oxygenases. Specifically, arachidonic acid is metabolized through three distinct pathways: cyclooxygenases (COX) to produce prostaglandins and thromboxanes, lipoxygenases (LOX) to produce hydroxyeicosatetraenoic acid (HETEs) and leukotrienes, and cytochrome P450 to produce epoxygenated fatty acids (EETs) (Smith, 1989). Since their discovery almost a century ago, this family of molecules have been implicated in almost every biological process including proliferation, differentiation, migration and invasion (Baker, 1990). In general, these molecules have short half-lives; they are produced in response to external stimuli such as growth factors or chemokines, and are released to act in either an autocrine or paracrine manner through binding to cell surface receptors. While the initial steps of eicosanoid production involve activation of intracellular phospholipases, production is mediated in a cell-specific fashion through a series of downstream synthases. Thus, the biological effects of eicosanoids in many diseases including cancer are context dependent, with distinct production by specific cell types signaling in turn to target cells.

In cancer, the role of eicosanoids has been examined for many years (Young, 1994; Wang and Dubois, 2010). Earlier studies focused largely on the role of prostaglandins, mainly prostaglandin E2 (PGE2) produced through the action of COX-1 or COX-2. Data from many investigators have shown that this eicosanoid is elevated in cancer. Studies demonstrated that PGE2 could act in an autocrine fashion to promote cell proliferation, migration and invasion (Sheng et al., 1998; Yip-Schneider et al., 2000; Raz, 2002). This resulted in the development of COX inhibitors, specifically COX-2 inhibitors as potential therapeutic agents in cancer (Jacoby et al., 2000; Csiki et al., 2005; Bertagnolli et al., 2006). While these agents had some efficacy, enthusiasm for targeting this pathway was diminished by the adverse side effects, specifically associated with risk for cardiovascular disease. Other eicosanoids were also considered pro-tumorigenic; however, their mechanism of action has not been well defined. Nevertheless, the consensus of these earlier studies was that eicosanoids in general promoted cancer progression, and these effects were mediated largely through direct effects on tumor cells.

The last 10 years has seen a significant “paradigm shift” from viewing cancer as a disease driven through activation of oncogenes and loss of tumor suppressor genes via somatic mutations, to a systemic disease involving an elaborate interplay between the transformed cancer cells and the surrounding microenvironment (Whiteside, 2008). These changes are reflected in the updated hallmarks of cancer defined by (Hanahan and Weinberg, 2011). In particular, one of these novel hallmarks is evasion of anti-tumor immunity. The focus on the ability of the immune system to inhibit, and potentially eliminate tumors has long been considered a potential therapeutic approach. However, the last 5 years has seen this come to fruition with the development of immunotherapy, specifically immune checkpoint inhibitors (Topalian et al., 2012; Topalian et al., 2015). These agents, which target pathways that cancer cells use to block anti-tumor effects of T cells have shown remarkable efficacy in a variety of malignancies, leading to FDA approval (Callahan and Wolchok, 2013; Gajewski et al., 2013). However, the overall response rate to this class of agents remains low (Camidge et al., 2019; Schoenfeld and Hellmann, 2020); thus a better understanding of mechanisms that control this response is required. Tumors are able to mobilize a variety of strategies to block anti-tumor T cells, and defining these pathways with a goal of developing rational combination therapies is being avidly pursued. In this context, it has become clear that specific eicosanoids have immunosuppressive activity. Thus, the role of these lipid mediators needs to be reexamined, beyond how they directly affect cancer cells, but also extending to an understanding of how they condition the tumor microenvironment. The prior development of both inhibitors and activators or eicosanoid pathways make these molecules attractive therapeutic targets (Narumiya et al., 1999). The goal of this review is to focus on the role of eicosanoids as modulators of the tumor microenvironment and specifically anti-tumor immunity. We will review published studies examining this, and also indicate where there are gaps in our knowledge regarding the role of this complex family in controlling immunity.

### Pathways of Eicosanoid Production

**FIGURE 1:**
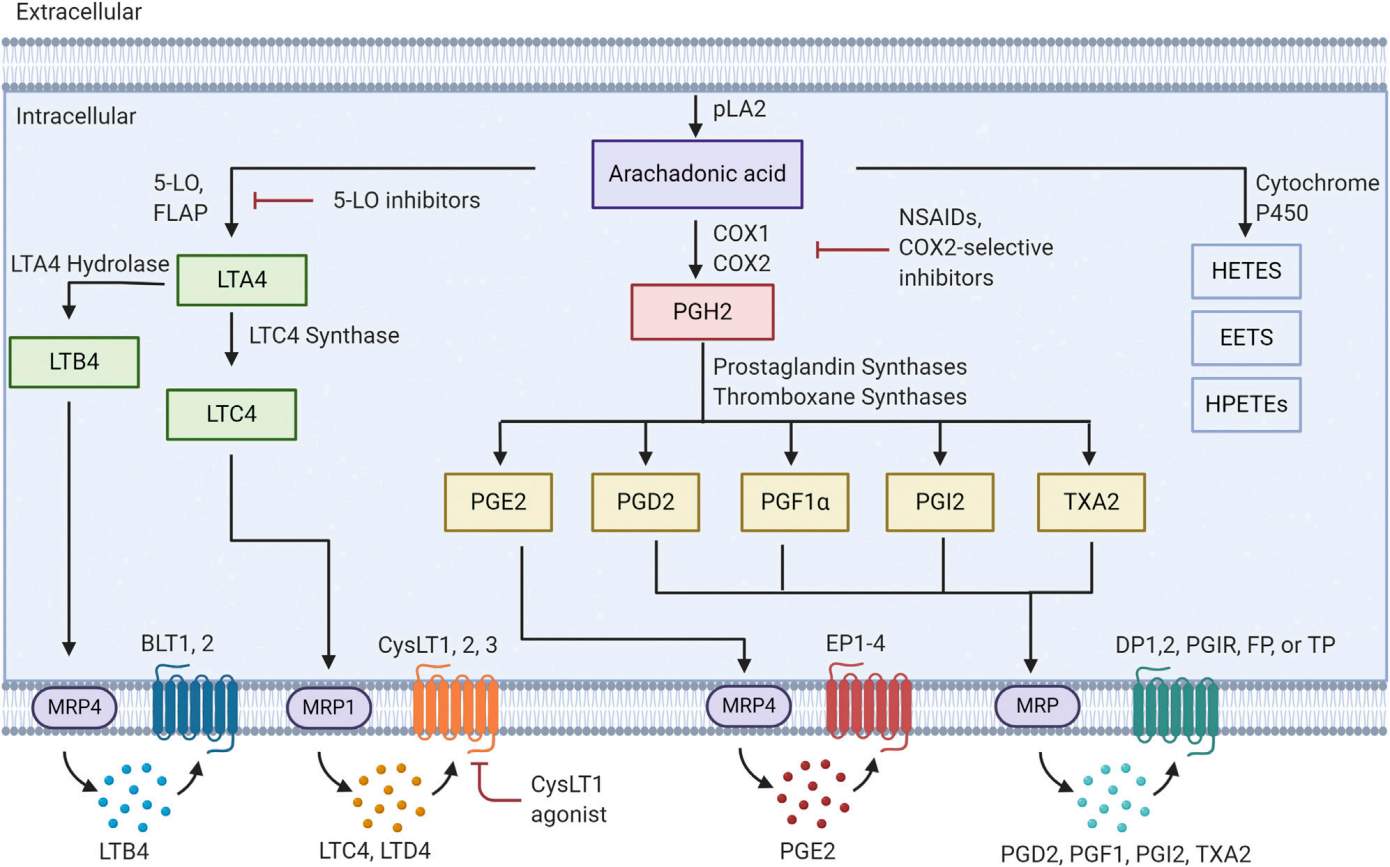
Eicosanoid pathway Activation of PLA2 results in release of arachidonic acid from membrane phospholipids. This free arachidonic acid can be metabolized to a family of over 30 distinct bioactive lipids. These molecules are released from cells and act in an autocrine or paracrine fashion by binding to specific receptors.

Since there are numerous extensive reviews on pathways mediating eicosanoid production (Cathcart et al., 2011; Castelino, 2012; Dennis and Norris, 2015), we will only provide an overview of these pathways. In most cells the majority of arachidonic acid (AA) is esterified into membrane phospholipids (Figure 1). Release of AA is mediated through the action of the phospholipase A_2_ (PLA_2_) family of enzymes, and this usually represents the rate limiting step in eicosanoid production (Nakanishi and Rosenberg, 2006). The major isoform of PLA_2_ implicated in regulated release of AA is Group IVA, designated cPLA_2_ (Gronich et al., 1990; Balsinde and Dennis, 1997). This enzyme has specificity for arachidonic acid-containing phospholipids, is ubiquitously expressed and in resting cells is generally located in the cytoplasm (Clark et al., 1990; Bonventre, 1992; Leslie, 1997). Stimulation of cells resulting in increased levels of intracellular Ca^2+^ results in translocation of cPLA_2_ to the membrane (Glover et al., 1995; Gronich et al., 1988). In most cells this translocation is to the nuclear envelope/endoplasmic reticulum (ER), which is the site of localization of many of the downstream effectors. In spite of extensive research over the past two decades, the factors that determine the site of localization are incompletely understood. However, the nuclear envelope/ER is the location of many of the downstream enzymes mediating AA release, including COX1/2 and 5-lipoxygenase (5-LO). While cPLA_2_ is ubiquitously expressed, the downstream synthases show much greater degrees of cell-specific expression, resulting in differential expression by different cells in the tumor microenvironment.

Prostaglandin production is catalyzed through expression of either cyclooxygenase-1 or -2 (COX-1/2). In general COX-1 is expressed more widely in many cell types. However, COX-2 is expressed at low levels in most cell types, and is transcriptionally induced in response to inflammatory stimuli. Prostaglandin H2 (PGH2), which is produced through the metabolism of AA by COX-1/2 can then lead to multiple prostanoid products: prostaglandin E2, prostaglandin D2 (PGD2), prostacyclin (PGI2), prostaglandin F1α (PGF1α) and Thromboxane A2 (TXA2), via expression of downstream synthases: PGE2 synthases (mPGES-1,2), PGD synthases, PGI synthase (PGIS) and thromboxane synthases (Seo and Oh, 2017). These lipid mediators are then exported out of the cell, via multiple eicosanoid transporters, where they can bind in an autocrine or paracrine fashion to cell surface receptors. There are four PGE2 receptors (EP1,2,3,4), two PGD2 receptors (DP1,DP2), a PGI2 receptor (PGIR), a PGF1α receptor (FP) and a thromboxane receptor (TP) (Wang and DuBois, 2018). The majority of these receptors are members of the G-protein coupled receptor family and engage downstream signaling pathways. Finally, breakdown of these products is mediated through specific enzymes such as hydroxyprostaglandin dehydrogenase 15-(NAD) (HPGD) (Hughes et al., 2008; Tai, 2011).

Leukotrienes are produced through the action of 5-lipoxygenase, and 5-lipoxygenase activating protein (FLAP). FLAP serves to present AA to the 5-lipoxygenase enzyme, resulting in production of leukotriene A4 (LTA4) (Kanaoka and Austen, 2019). Subsequent metabolism of this product occurs through LTA4 hydrolase to produce leukotriene B4 (LTB4), or leukotriene C4 synthase (LTC4 synthase) to produce LTC4. LTC4 is subsequently exported and can be converted to leukotriene D4 (LTD4) through the actions of γ-glutamyl transpeptidases, and leukotriene E4 (LTE4) via membrane bound dipeptidases (Yokomizo et al., 2018). Specific G-protein coupled receptors for these products have been identified, with both a high and low affinity receptor for LTB4 (BLT1, BLT2) and two receptors for products of LTC4 synthase (CystLT1 and CystLT2) (Yokomizo et al., 2018). Antagonists for these receptors have been developed and the CystLT1 antagonists montelukast and zafirlukast are approved to treat bronchial asthma and allergic rhinitis (Capra et al., 2006). Recently a third receptor, designated as CystLT3 or GRP99 has been identified (Kanaoka et al., 2013). This receptor appears to be specific for LTE4.

Finally, the cytochrome P450 pathway results in the production of epoxyeicosatrienoic acids and the hydroxyeicosastetraeinoic (Chen and Wang, 2015). These molecules have been shown to have vasculo-protective effects through mediating vasodilation and angiogenesis (Evangelista et al., 2020). The role of these molecules in cancer has not been as well studied as the other classes of eicosanoids.

In addition to signaling through cell surface receptors, numerous eicosanoids can also signal by binding to and activating specific nuclear receptors of the peroxisome proliferator-activated receptor family (PPARs) specifically the peroxisome proliferator activated receptor family PPARγ or PPARδ (Berger and Moller, 2002; Nixon et al., 2003). In particular, the role of PPARγ has been extensively studied in multiple types of cancer (Berger and Moller, 2002; Grommes et al., 2004; Panigrahy et al., 2005; Nemenoff, 2007; Roman, 2008). While the role of these receptors will not be discussed in this review, it should be noted that the ability of eicosanoids to engage these receptors on different cell types adds to the complex biology of these mediators.

### Regulators of Tumor Immunity

Pathways of immune evasion: The advent of immunotherapy has radically changed how lung cancer is treated, and altered the focus of preclinical research. While the ability of the immune system to recognize tumors has been appreciated for a long time, immune evasion was only recently defined as one of the hallmarks of cancer (Hanahan and Weinberg, 2011). A conceptual model to describe cancer-immune interactions is the immunoediting hypothesis. In this model, cancer cells develop a variety of strategies to avoid elimination by the host immune system (Schreiber et al., 2011). Initial recognition of cancer cells by the immune system is followed by adaptation of the cancer cells, leading to an equilibrium phase, and subsequently escape and cancer progression. Tumors have developed a variety of strategies to avoid T cell-mediated killing of cancer cells. These include loss of antigen presentation, through changes in MHC expression, or accessory pathways required for peptide binding (Pardoll, 2012; Gajewski et al., 2013), as well as changes in the relative populations of specific cell types comprising the tumor microenvironment, such as increases in immunosuppressive myeloid suppressor cells and tumor-associated macrophages (Mantovani et al., 2007; Whiteside, 2008; DeNardo et al., 2010). Based on these studies, it has been proposed for some time that therapeutic strategies targeting these pathways, and resulting in reestablishment of anti-tumor immunity would result in tumor elimination. Furthermore, these strategies could result in the formation of memory T cells, thus affording long term efficacy for these treatments. While initial attempts at immunotherapy were unsuccessful, recent studies have developed multiple strategies which have shown long-lasting effects in many cancers, resulting in a “immunotherapy revolution” in cancer (Topalian et al., 2015; Rothschilds and Wittrup, 2019).

Prominent among these pathways are immune checkpoints which are mechanisms designed to regulate the extent and the duration of the immune response critical for preventing autoimmune responses. Cancer cells have adopted these pathways to inactivate infiltrating T cells. The most well studied immune checkpoints are the CTLA4 and PD1/PD-L1 pathways (Pardoll, 2012; Gajewski et al., 2013). The PD-1/PD-L1 pathway involves upregulation of PD-L1 on cancer cells and other stromal cells, which binds to PD-1 expressed on activated T cells. This interaction inhibits T cell function resulting in what has been termed an “exhausted” phenotype. Similarly, CTLA4 expressed on T cells is bound by CD80 and CD86 expressed on cancer cells to inhibit T cell activation (Pardoll, 2012). Monoclonal antibodies targeting these interactions (anti-PD-1 or PD-L1, or anti-CTLA4) block these interactions, resulting in reactivation of T cells, and tumor elimination. These agents have shown surprising efficacy in a number of cancers including melanoma, non-small cell lung cancer (NSCLC), and head and neck squamous cell carcinoma, leading to current FDA approval for the treatment of 14 distinct malignancies, and the awarding of the Nobel Prize to the discoverers of this pathway. However, only approximately 20% of unselected cancer patients show a long-lasting response to this therapy (Doroshow et al., 2019; Havel et al., 2019; Tang et al., 2018). Thus, a major unmet need is to define the cellular and molecular mechanisms that determine the responsiveness to immune checkpoint inhibitors, and to develop rational combinations to increase patient responses.

The determinants mediating response to immunotherapy remain poorly understood. There clearly is an association of response to immunogenic burden and neoantigens (Rizvi et al., 2015). However, even in patients with high mutational burden there is a heterogeneity of response to checkpoint inhibitors. Immunosuppression can be mediated by a variety of mechanisms. In addition to immune checkpoints, recruitment of regulatory T cells (Treg) or myeloid derived suppressor cells (MDSC) can inhibit cytotoxic T cells independent of checkpoint pathways (Veglia et al., 2018; Togashi et al., 2019). Recently, Gajewski and coworkers have determined that in melanoma, patients that are resistant to checkpoint inhibitors have very few infiltrating T cells, designated an “uninflamed” tumor, compared to responders which have an “inflamed” phenotype comprising abundant infiltrating T cells (Gajewski et al., 2013). In their model, inherent properties of the cancer cells, distinct from mutational burden signal to the TME to limit T cell infiltration and interactions with cancer cells. Defining these pathways will be critical in allowing an increase in the response rate to immunotherapy. Other inherent properties of the cancer cell have also been associated with responsiveness. Ayers et al. have developed a gene signature involving responsiveness to interferon gamma (IFNγ) associate with response (Ayers et al., 2017). Our lab has demonstrated that at least in mouse models of lung cancer responsiveness to IFNγ is a determinant of response to anti-PD-1 therapy (Bullock et al., 2019).

Following the success with a single immune checkpoint inhibitor, a large number of clinical trials are currently examining combinations of immune checkpoints (e.g., anti-CTL4+antiPD-1) in multiple malignancies (Tang et al., 2018). While the response rate to these combinations is increased, there are still large numbers of patients that are resistant, and there is an increase in cytotoxicity in many of these trials. In addition to immune checkpoint inhibitors, many other immunosuppressive pathways have been identified which allow cancer cells to evade immune attack. These include increases in regulatory T cells (Treg) (Togashi et al., 2019), loss of antigen presentation (Johnson et al., 2018), (Rosenthal et al., 2019), or altered IFN signaling (Wang et al., 2016). Of particular interest is the role of innate immunity in mediating immunosuppression. Innate immune cells comprise multiple subtypes, including neutrophils, monocytes/macrophages, myeloid derived suppressor cells and others. In addition to interacting with resident innate immune populations, cancer cells produce chemokines that attract specific populations of circulating myeloid cells. While many of these populations initially are mobilized to inhibit tumor growth, cross-talk between cancer cells results in phenotypic conversion of these populations. For example, monocyte/macrophages recruited to tumors have been shown to manifest a classic inflammatory phenotype, designated as M1, which can inhibit cancer progression (Mantovani et al., 2002; Murray et al., 2014). However, interactions with tumor cells can alter this phenotype and promote a phenotype associated with resolution of inflammation and wound healing, designated M2 (Sica et al., 2007).

### Eicosanoids and Cancer

**FIGURE 2:**
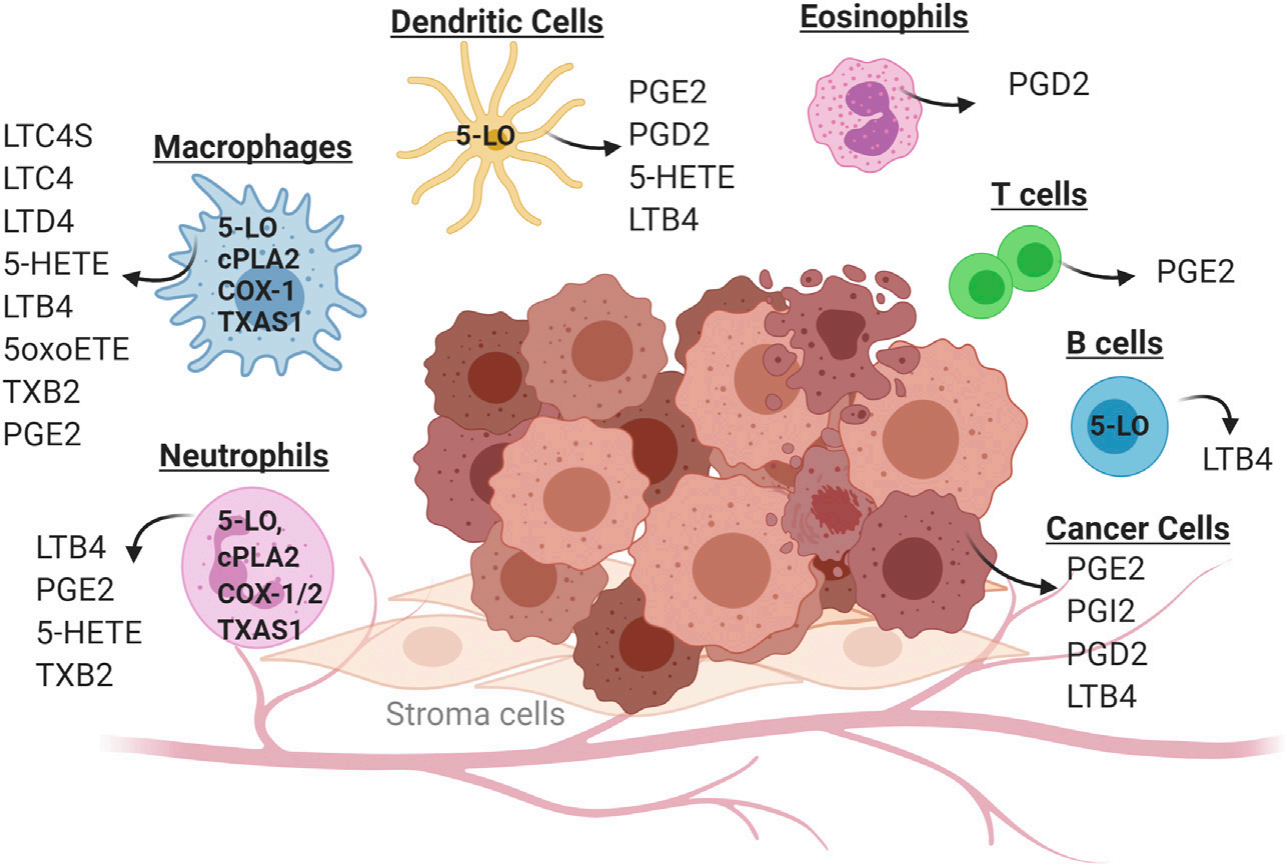
Cell-specific production of eicosanoids in the tumor microenvironment. Solid tumors consist of cancer cells (transformed epithelial cells) in close contact with a non-transformed cells of the tumor microenvironment. These include inflammatory cells (neutrophils, macrophages, eosinophils and dendritic cells) as well as cells of the adaptive immune system (T cells and B cells). Individual cell types in the tumor microenvironment produce distinct panels of eicosanoid as a consequence of cell-specific expression of enzymes in this pathway. Some eicosanoids are produced by multiple cell types, such as PGE2 which can be produced by cancer cells, macrophages and neutrophils.

Studies dating back at least 40 years have shown that eicosanoids play a critical role in both cancer initiation and progression (Kisley et al., 2002; Edelman et al., 2008; Horn et al., 2009; Wang and Dubois, 2010; Cathcart et al., 2011). Production of eicosanoids has been shown to promote growth of cancer cells in an autocrine fashion by signaling through cell surface receptors (Tong et al., 2005; Nie, 2007; Moreno, 2009; Wang and Dubois, 2010; Cathcart et al., 2011; Schneider and Pozzi, 2011). Earlier studies have demonstrated that eicosanoids can control multiple biological processes in the setting of cancer, including cell proliferation, migration, and differentiation (Wang and Dubois, 2010). While these studies have focused on eicosanoids produced by cancer cells, our data and those of many others indicate that while tumor cells predominantly produce PGE2, the TME produces a broad spectrum of eicosanoids (Kudryavtsev et al., 2005; Greene et al., 2011; Panigrahy et al., 2011; Poczobutt et al., 2013; Moore and Pidgeon, 2017) (see Figure 2). These products are produced in large part by inflammatory cells, but act on other cell types, particularly cells of the adaptive immune system (CD4^+^ and CD8^+^ T cells). These observations increase the complexity of studying the role of individual eicosanoids, since it is necessary to consider the specific cell producing these agents, as well as the specific product produced. In fact, it is likely that the role of a particular product may differ depending on where and when it is produced during cancer progression. In the following sections, we will focus on eicosanoids that act on immunosuppressive pathways.

### Role of Prostaglandins

The most studied prostaglandin is PGE2, produced mainly through the sequential action of COX-2 and microsomal PGE-synthase (mPGES-1). While earlier studies focused on the direct effects of PGE2 on cancer cells, there is emerging data indicating that this eicosanoid has important immunosuppressive pathways (Wang and DuBois, 2018). In many cases, PGE2 acts on innate immune cells to alter the tumor microenvironment. In pancreatic cancer, induction of COX-2, which is mediated through the ephrin A receptor two results in tumors in which T cells are largely excluded (Markosyan et al., 2019), and there are abundant increases in the numbers of myeloid derived suppressor cells. As such, these tumors are resistant to immune checkpoint inhibitors. Markosyan and co-workers demonstrated that genetic or pharmacologic inhibition of COX-2 alters the TME, resulting in infiltration of T cells, and decreases in MDSC, suggesting that combinatorial therapy with COX-2 inhibitors such as celecoxib and anti-PD-1 may increase response (Markosyan et al., 2019). MDSCs act to suppress the anti-tumoral activity of T cells and other cell types (Veglia et al., 2018). In a lung cancer model in which Gprc5a is deleted, increased PGE2 synthesis was also associated with recruitment of MDSCs as well as promotion of alternatively activated M2 macrophages (Wang et al., 2020). PGE2 has been shown to signal through the EP2 receptor to induce an immunosuppressive phenotype in MDSC (Porta et al., 2020). These effects are mediated through nuclear accumulation of p50 NF-κB, and production of nitric oxide (NO). Recent data indicate that PGE2 can alter the phenotype of immune cells through regulation of epigenetic mechanisms. Specifically, PGE2 produced by cancer cells can act on myeloid cells to induce DNA methyltransferase 3A (DNMT3A), leading to alterations in DNA methylation and suppression of immunogenic genes (Rodríguez-Ubreva et al., 2017).

A number of additional mechanisms for the immunosuppressive effects of PGE2 have been proposed, and these appear to be cancer-type specific. Earlier studies showed that PGE2 collaborates with TGF-β to increase Tregs (Baratelli et al., 2005; Baratelli et al., 2010). More recently, PGE2 production has been implicated in directly mediating resistance to immune checkpoint inhibition (Zelenay et al., 2015). Induction of PD-L1 expression in tumor associated macrophages is mediated through PGE2 (Prima et al., 2017). PGE2 has been shown to induce expression of cell-surface ectonucleases CD39 and CD73 on CD14^+^ monocytes (Al-Taei et al., 2017), resulting in production of adenosine is a breakdown product of ATP that mediates tumor progression and immunosuppression. PGE2 acting through the EP4 receptor has been shown to induce the immune checkpoint protein TIM3 on Jurkat T cells (Yun et al., 2019). Mesenchymal stem cells (MSC) from tumors selectively inhibited the function of NK cells in tumors, and this is mediated by PGE2 production by the MSC (Galland et al., 2017).

Dendritic cells are critical for engaging the adaptive immune system in inhibiting cancer progression. While distinct populations of dendritic cells have been defined, conventional dendritic cells (cDC) present tumor antigens to T cells, as well as produce cytokines to regulate T cell effector function (Wculek et al., 2020). While factors regulating recruitment of dendritic cells to tumors, data support a role for natural killer cells (NK) in cDC1 recruitment (Böttcher et al., 2018). PGE2 production by tumor cells inhibits this recruitment by impairing NK viability of cytokines production (Böttcher et al., 2018). The mechanisms responsible for inducing this population are not well understood. Recent data has demonstrated increased lipid accumulation in neutrophils mediated through fatty acid transporter 2 (Veglia et al., 2019) results in their promotion of immunosuppression. This is mediated at least in part through increased production of PGE2.

In colon cancer, PGE2 derived through COX-2 has been shown to lead to a feed forward mechanism, in which receptor-interacting protein kinase 3 (RIPK3) results in activation of myeloid suppressor cells and suppression of anti-tumor immunity (Yan et al., 2018). PGE2 leads to down-regulation of RIPK3, which in turn results in induction of COX-2 and increased production of PGE2 by the MDSCs, which in turn increases proliferation of cancer cells and inhibits the anti-tumor function of CD8^+^ T cells.

The existence of four PGE2 receptors which signal through different pathways increases the complexity of developing effective therapeutic agents. Antagonists have been developed to target individual receptors. Antagonists against EP4, which signals through cAMP, have shown efficacy in multiple types of cancer (Majumder et al., 2018; Ching et al., 2020). EP3 antagonists have been shown to be effective in inhibiting growth of breast cancer cells (Hester et al., 2019), whereas EP1 antagonists are effective in models of hepatocellular carcinoma (Yang et al., 2019). The role of individual receptors in mediating immunosuppression has not been extensively examined, but these agents are likely to be more selective than broad inhibition of PGE2 production.

PGD2, in contrast has been shown to inhibit induction of expression of elevated levels of Indoleamine 2,3-dioxygenase (IDO) by macrophages (Bassal et al., 2016). Since IDO is associated with immunosuppression, these data would suggest that PGD2 would inhibit immunosuppression by blocking production of tryptophan metabolism (Bassal et al., 2016). However, PGD2 has also been shown to foster immunosuppression in acute promyelocytic leukemia through modifying Group 2 innate lymphoid cells and MDSCs (Trabanelli et al., 2017); these authors also show that strategies to decrease PGD2 relieve this immunosuppression.

In contrast to the general pro-tumorigenic effects of PGE2, prostacyclin produced through the action of prostacyclin synthase has been shown to inhibit both tumor initiation and progression (Keith et al., 2002; Keith et al., 2004; Li et al., 2018). Genetic mice with targeted overexpression of PGIS in the lung were protected against induction of lung tumors in response to either chemical carcinogenesis or exposure to cigarette smoke (Keith et al., 2002; Keith et al., 2004). While the mechanisms for these effects are not understood, immunostaining revealed alterations in macrophage staining in response to elevated prostacyclin. A clinical trial examining the effect of the prostacyclin analog iloprost in patients at risk for developing lung cancer demonstrated reduced progression of preneoplastic lesions in former smokers (Keith et al., 2011), underscoring the potential of prostacyclin analogs as chemopreventive agents. Elevated prostacyclin also inhibits tumor growth in mouse models of lung cancer (Li et al., 2018). This was mediated by increased numbers of CD4^+^ T cells into the tumors. In other systems it has been shown that PGI2 regulates CD4^+^ T cell populations, and can inhibit the development of Tregs (Aronoff et al., 2007; Li et al., 2018).

### Role of Leukotrienes

Leukotrienes produced through the action of 5-lipoxygenase have been detected in both cancer cells and cells of the TME. In cancer cells, the majority of the published data support a pro-tumorigenic role for these lipid mediators, through direct effects on cell proliferation (Rioux and Castonguay, 1998; Steinhilber et al., 2010; Merchant et al., 2018). However, the role of leukotrienes in the TME appears to be more complex. While some cancer cells can produce these lipids, leukotrienes are mainly produced by inflammatory cells, including neutrophils, macrophages, and eosinophils (Steinhilber et al., 2010; Wang and Dubois, 2010; Esser-von Bieren, 2017; Merchant et al., 2018). Studies in our lab using an immunocompetent model of lung cancer, demonstrated that resident macrophages are the highest expressers of 5-lipoxygenase (Poczobutt et al., 2016a).

Several studies have demonstrated that leukotriene production by tumor associated macrophages is critical for T cell recruitment. In particular, LTB4, produced through the actions of 5-lipoxygenase and LTA4 hydrolase, has been shown to be a potent chemotactic factor for the recruitment of T cells (Goodarzi et al., 2003; Jala et al., 2017a; Jala et al., 2017b; Tager et al., 2003). While other factors, specifically ligands which bind to CXCR3 on T cells, such as CXCL9 and CXCL10 are critical for T cell recruitment to tumors, deletion of BLT1 and CXCR3 result in equal impairments in T cell recruitment, and the double knockout mice do not show any additivity, suggesting that both of these pathways are required (Chheda et al., 2016). Consistent with these data, apoptotic cancer cells have been shown to reduce expression of 5-lipoxygenase in tumor associated macrophages, and this is associated with decreased T cell recruitment and tumor progression (Ringleb et al., 2018). Interestingly, the response of melanoma tumors to immune checkpoint inhibitors such as anti-PD-1 is abrogated if these tumors are growing in BLT1-deficient mice (Chheda et al., 2016). These data indicate that administration of analogs of LTB4 may augment the response to immune checkpoint blockade. This needs to be confirmed in relevant preclinical models. Furthermore, examination of LTB4 and its role in T cell recruitment needs to be examined in human tumors. A clinical trial using an LTB4 antagonist which was expected to inhibit lung cancer progression by acting on the tumor cells, actually resulted in worsening of disease (Jänne et al., 2014), suggesting that this agent is targeting the TME. Based on the preclinical data, it might be anticipated that this agent would decrease T cell recruitment to tumors, thus promoting progression.

Studies using the APC^min/+^ mouse, a model for colorectal cancer demonstrate the complexity of this pathway. Initial studies using 5-lipoxygenase inhibitors, resulted in inhibition of tumor progression (Mohammed et al., 2011). However, more recent studies using mice deficient in expression of the LTB4 receptor BLT-1 (APC^min/+^/BLT^−/−^), resulted in increased tumor growth, suggesting an inhibitory role for this eicosanoid (Jala et al., 2017a; Jala et al., 2017b). Further, in APC^min/+^ mice deficient in 5-lipoxygenase (APC^min/+^/5-LO^−/−^) tumor growth was inhibited. This is likely due to pro-tumorigenic effects of other 5-LO products, such as LTC4. Tumor-associated macrophages show a decreased expression for 5-lipoxygenase which appears to be mediated through direct cell-cell contact and MerTK signaling (Ringleb et al., 2018). These authors suggest that 5-LO may be marker for tumor associated macrophages (TAMs).

In fact, in colon cancer leukotrienes have been linked to a number of other hallmarks of cancer, including angiogenesis and altered metabolism (Burke et al., 2016). In ovarian cancer, 5-lipoxygenase products act to recruit tumor associated macrophages (Wen et al., 2015) and expression was associated with metastasis. These effects were likely mediated at least in part through regulation of MMPs. Since the role of eicosanoids are context dependent, additional studies are needed in other types of cancer (Wen et al., 2015).

In addition to binding to its cognate receptor, LTB4 can activate nuclear receptors, specifically peroxisome proliferator activated receptor-α (PPARα) (Devchand et al., 1996). While the role of this family of receptors in cancer is complex, and beyond the scope of this review, activation of PPARα by LTB4 in B cells promotes the generation of regulatory B cells (Breg) and promotes breast cancer metastasis (Wejksza et al., 2013). A role for leukotrienes has also been demonstrated in metastasis. Production of leukotrienes promotes formation of metastasis-initiating cells (MIC) in a breast cancer model (Wculek and Malanchi, 2015). These cells express leukotriene receptors, and in turn regulate the colonization and expansion of cancer cells in the premetastic niche.

These studies suggest potentially opposing effects of different 5-lipoxygenase products on cancer progression and immunity. Unfortunately, other than LTB4, there have not been extensive studies on the specific roles of the other leukotrienes (LTC4, D4 and E4). The existence of genetic mice which lack these products should be a valuable tool in examining the role of this pathway in greater detail.

### Cytochrome P450 Metabolites

While there have been fewer studies examining these products, strong data indicate that EETs, and HETEs play an important role in tumor angiogenesis, and thus are likely to regulate anti-tumor immunity indirectly (Moreno, 2009; Panigrahy et al., 2011; Panigrahy et al., 2012; Chen and Wang, 2015). Cancer cells as well as cells of the TME have been shown to produce these products. Decreased production in lung cancer cells has been associated with slower disease progression (Sausville et al., 2018). There is very little known regarding the direct role of these products on either innate or adaptive immunity (Evangelista et al., 2020).

## Conclusion and Future Directions

The role of eicosanoids in cancer has been studied for many years, with publications as early as the 1960s (Hydovitz, 1968; Grimley et al., 1969). Initially the focus of these lipid mediators was on direct effects on cancer cells, focusing on proliferation and migration. This culminated in the development of selective COX-2 inhibitors as potential therapies for multiple types of cancer. However, the complications associated with these agents decreased enthusiasm for targeting eicosanoid pathways in cancer, and a decrease in the effort to define these pathways. More recently, with the explosion of interest in the tumor microenvironment, there has been a renewed interest in the role of eicosanoids in regulating the immune response and altering the composition of the TME. These studies have established a role of both products of the COX and 5-LO pathway in regulating anti-tumor immunity. Many of these studies indicate an immunosuppressive role for these lipid mediators, distinct from their direct effects on cancer cells. Eicosanoids have been shown to modify the anti-tumor effects of cytotoxic T cells (DiMeo et al., 2008; Poczobutt et al., 2016b), alter the populations of innate immune cells to favor increases in immunosuppressive cells such as MDSC and tumor associated macrophages (Lone and Tasken, 2013; Esser-von Bieren, 2017), as well as modulating metabolic pathways such as IDO1 (Yang et al., 2001; Moore and Pidgeon, 2017). Thus, inhibitors of this pathway are attractive agents to use in combination with approved immunotherapies such as checkpoint inhibitors. Nevertheless, there are still important gaps in our understanding of eicosanoids in the TME.

From the experience with COX-2 inhibitors, it appears likely that systemic blockers of eicosanoid pathways will have undesirable side effects, limiting their utility as anti-cancer agents. Therefore, a potential strategy is to selectively target the action of specific eicosanoids on specific cell populations. This is well-illustrated in the case of leukotrienes. These mediators appear to promote cancer cell proliferation, and there have been numerous preclinical studies using inhibitors of 5-lipoxygenase demonstrating inhibitory effects on cancer progression (Rioux and Castonguay, 1998; Pace et al., 2004; Tong et al., 2005). However, since these molecules affect T cell recruitment into tumors, systemic blockade of this pathway may result in tumors with fewer infiltrating T cells, and this would be expected to block the action of immunotherapies such as checkpoint inhibitors (Rioux and Castonguay, 1998; Pace et al., 2004; Tong et al., 2005; Sharma et al., 2013; Satpathy et al., 2015; Chheda et al., 2016; Poczobutt et al., 2016). It is not known if these disparate effects are mediated through production of leukotrienes in different cell types, or whether the products produced by a specific cell type have opposing effects (Moore and Pidgeon, 2017). An additional complication is that targeting one arm of the eicosanoid pathway may result in promoting increased flux through another branch. For example, it has been shown that in some settings blocking 5-lipoxygenase activity will result in greater conversion of arachidonic acid through the COX pathway, resulting in increased prostaglandin production (Kudryavtsev et al., 2005). A more selective approach would involve blocking downstream targets of these enzymes targeting either the specific synthases or the receptors (see Figure 2). The majority of these receptors are G-protein coupled receptors, and these have been historically amenable to inhibition by small molecules. However, there are only a limited number of studies that have tested these compounds in relevant models of cancer, and their effects on the immune response to cancer is not well studied.

Finally, additional translational studies are required. Eicosanoids were originally thought to represent attractive targets for cancer therapy. However, the adverse effects associated with COX-2 inhibitors diminished the enthusiasm for targeting this pathway, which persists to some degree to this day. The advent of immunotherapy and the focus of targeting anti-tumor immunity has been a paradigm shift in cancer therapeutics and has provided new hope for many cancer patients. This has resulted in an explosion of clinical trials using combinations of immune checkpoint inhibitors and other therapies, often with little scientific rationale (Tang et al., 2018). Selectively targeting eicosanoid pathways has a strong basis in preclinical studies, and well-designed trials accompanied by analysis of tumor samples may lead to a renewed enthusiasm for these pathways. In addition, the availability of selective agonists and antagonists acting on specific eicosanoid receptors offer an opportunity to target specific biological roles of these molecules without the complications entailed in blocking entire pathways.

## Author Contributions

AJ prepared the figures and helped write the manuscript. EK helped write the manuscript. RN oversaw the manuscript and wrote the manuscript.

## Funding

This work was supported by grants from the NIH (R01 CA162226 and P50 CA058187), LUNGevity Foundation and the Cancer League of Colorado. AJ was supported by the NIH NRSA T32 CA174648-01. Figures were created with BioRender.com.

## Conflict of Interest

The authors declare that the research was conducted in the absence of any commercial or financial relationships that could be construed as a potential conflict of interest.
